# An Acute Abdominal Catastrophe in a HIV Positive Patient

**DOI:** 10.4021/gr451w

**Published:** 2012-05-20

**Authors:** Vinaya Gaduputi, Harish Patel, Vamshidhar Vootla, Usman Khan, Sridhar Chilimuri

**Affiliations:** a Bronx Lebanon Hospital Center, Department of Medicine, 1650 Selwyn Ave, Suit #10C, Bronx, New York 10457, USA

**Keywords:** Acute abdomen, HIV

## Abstract

We report this case of a 45-year-old man with HIV-AIDS on HAART therapy who presented with acute abdominal pain and renal failure. He was found to have pneumatosis intestinalis on computerized axial tomography scan of the abdomen. He underwent emergent explorative laparotomy, which revealed a necrotic large bowel segment for which a right-sided hemicolectomy and ileostomy were performed. The patient subsequently developed septic shock and hypoxic respiratory failure. He expired a week after the surgical procedure. Acute abdominal events due to vascular catastrophes secondary to hypercoagulability, endothelial dysfunction and accelerated atherosclerosis have been reported in HIV positive patients.

## Introduction

Longevity of the patients infected with HIV has increased since the introduction of Highly Active Antiretroviral Therapy (HAART). However, many patients on HAART therapy develop long-term metabolic derangements such as dyslipidemia, diabetes mellitus and insulin resistance [1-3]. HAART therapy and HIV infection in itself, produce chronic inflammation and cellular homeostatic stress response cascade [4] .It has been postulated that the increased cardiovascular risk is the result of HIV induced direct viral injury, endothelial dysfunction, hypercoagulabilty [5, 6]; opportunistic infections induced vaso-occlusive disease (especially CMV) [7] or chronic inflammatory state [8]. HIV induced large-vessel related catastrophes have been reported in the literature, including acute abdomen resulting from intestinal ischemia. The review of literature has shown this to happen usually as a result of atherogenic lipid profile (induced by HIV, HAART therapy or both) [9] or direct vaso-occlusive effect of opportunistic infections such as CMV. We postulate that subclinical atherosclerosis (as observed in Carotid and Coronary Intima media thickening (IMT) measurement studies [10] of the mesenteric arterial tree with superimposed hypercoagulabilty has a potential to produce critical ischemia, in presence of a precipitating cause such as renal failure or sepsis. We present one such unusual presentation of mesenteric ischemia probably secondary to such atherosclerosis.

## Case Report

**Figure 1:**
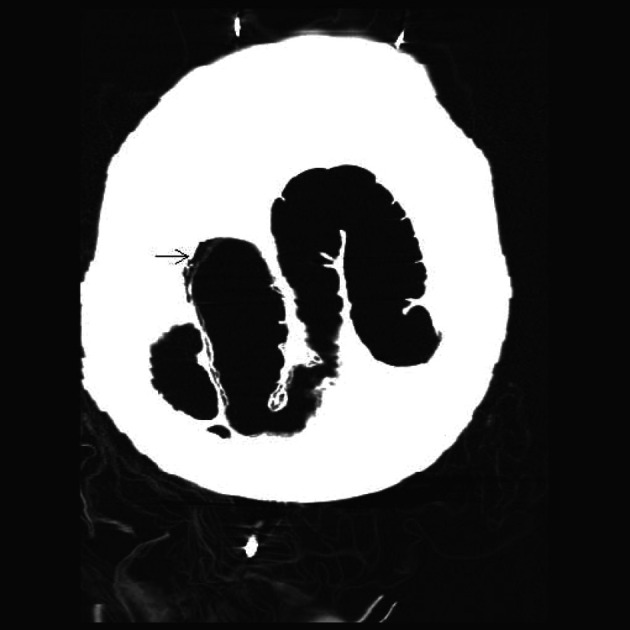
Coronal section of a Non-Contrast CAT scan of the abdomen revealing mural gas lucencies (black arrow) in the cecum, ascending and proximal portion of the transverse colon suggestive of extensive bowel necrosis.

**Figure 2:**
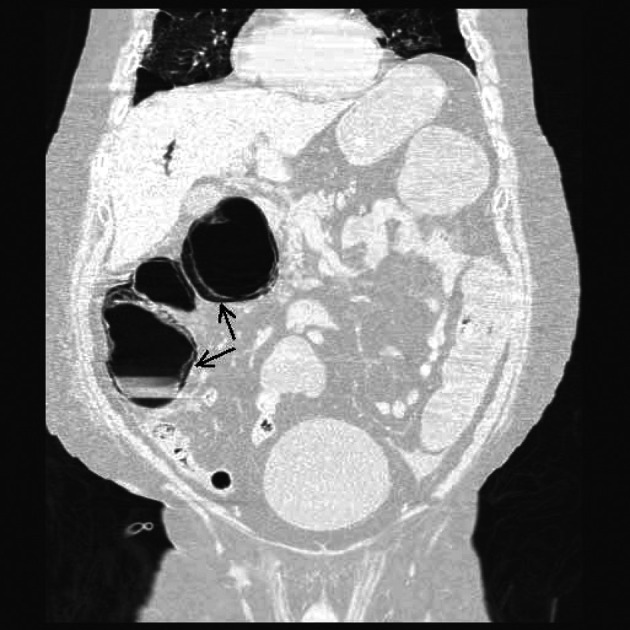
Coronal section of a Non-Contrast CAT scan of the abdomen revealing mural gas lucencies (white arrows) in the cecum, ascending and proximal portion of the transverse colon suggestive of extensive bowel necrosis.

**Figure 3:**
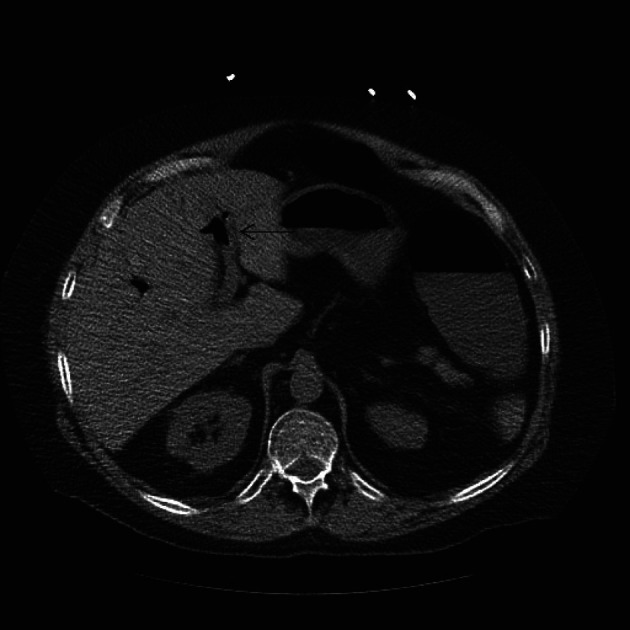
Transverse Section of a non-contrast CAT scan of abdomen showing the air bubble in the portal vein.

A 56-year-old Hispanic man with HIV-AIDS, hypertension and deep vein thrombosis (DVT) on Warfarin presented to the Emergency Room with severe midline low back pain and right sided flank pain. Physical examination revealed stable vital signs, oral thrush and unremarkable cardiopulmonary, abdominal examination. The computerized axial tomography (CAT) scan of the abdomen revealed no acute intra-abdominal pathology. However, CAT scan revealed osteoporosis with central endplate depression of L5 vertebra. Magnetic Resonance Imaging (MRI) of the lumbosacral spine failed to reveal any acute pathology but did show degenerative changes, bridging osteophytes. The patient was treated for musculoskeletal pain with opiate analgesics and oral thrush with Fluconazole, while continuing the HAART therapy. Subsequently, patient developed acute renal failure on the third day of hospitalization (serum creatinine increased from 1.3 mg/dL to 2 mg/dL) which worsened further acutely over the course of the next 24 hours (serum creatinine increased from 2 mg/dL to 7.9 mg/dL) with development of severe hyperkalemia (6.4). Patient’s HAART therapy and other nephrotoxic medications (Lisinopril, Allopurinol) were held. On the fourth day of hospitalization, patient developed diffuse abdominal pain and worsening constipation. A Computerized Tomogram of the abdomen revealed signs of bowel obstruction, pneumatosis intestinalis and free air in the mesenteric and portal venous systems (Fig. 1-3). The patient underwent emergent explorative laparotomy that revealed necrotic bowel that required right-sided hemicolectomy and ileostomy. Patient subsequently developed septic shock and died on the thirteenth day of hospitalization.

## Discussion

We present a middle-aged man with history of HIV-AIDS who presented with an acute mesenteric ischemia. Differential diagnoses of such presentation include: 1) mesenteric ischemia from an acute thrombotic event; 2) mesenteric ischemia resulting from vasocclusive opportunistic infections such as CMV; 3) mesenteric ischemia from an atheroembolic event; 4) an undiagnosed thrombophilia; 5) Non-occlusive mesenteric ischemia (NOMI) and 6) subclinical atherosclerosis with superimposed acute systemic insults such as renal failure and sepsis. Mesenteric ischemia from an acute thrombotic event as result of hypercoagulabilty is possible in HIV positive patients. Patient already had an episode of venous thrombosis in the past and was on an anticoagulation medication. The patient at the time of presentation was adequately anticoagulated. Mesenteric ischemia from an acute thrombotic event or an undiagnosed thrombophilia being the cause of this presentation is extremely unlikely in the setting of supra-therapeutic INR (5.5) of the patient. Opportunistic infections such as CMV would also have been unlikely, as patient’s CD4 T-helper cells count was above 150.

Though Protease Inhibitors are notorious for causing lipid panel anomalies and endothelial dysfunction [4], an atheroembolic cause is not probable in the setting of patient’s near normal lipid panel. A more direct evidence of this came from surgical pathology where the mesenteric vessels were found to be atherosclerotic but did not reveal any occlusive atheroma or thrombus. Also, there were no other evidences of metabolic syndrome or lipodystrophy seen with chronic protease inhibitor therapy. Nonocclusive mesenteric ischemia often presents with watershed zone ischemia. However, it is usually associated with hypovolemia, episodes of hypotension and precipitating drugs such as diuretics or vasoconstrictors, none of which were present in this patient [11]. However, NOMI has been found to be often associated with renal failure, such as found in this patient. Subclinical atherosclerosis produced by direct viral injury or metabolic effects of the HAART, superimposed by acute splanchnic vasoconstriction in response to acute renal failure and sepsis could have produced the clinical scenario as seen in this patient. Given the fact that he had acute renal failure, which is associated with NOMI and was on HAART therapy for HIV, which is known to be atherogenic as demonstrated by his atherosclerotic mesenteric vessels, mesenteric ischemia might have resulted from a combination of both the etiologies. With increasing use of HAART medications and increasing prevalence of resultant metabolic derangements, mesenteric ischemia should be seriously considered in patients presenting with acute abdomen.
